# Evaluation of musculoskeletal tumors in the new era of artificial intelligence

**DOI:** 10.1590/0100-3984.2018.51.6e3

**Published:** 2018

**Authors:** Flávia Martins Costa

**Affiliations:** 1 Clínica de Diagnóstico Por Imagem (CDPI) e Alta Diagnóstico - DASA, Rio de Janeiro, RJ, Brazil. E-mail: flavia26rio@hotmail.com. https://orcid.org/0000-0001-8070-3993.

Since the beginning of the 20th century, great progress has been made in the diagnosis and treatment of musculoskeletal tumors, contributing to a significant improvement in survival. That progress has resulted in the development of an integrated, multidisciplinary approach to patients and the advancement of many medical specialties, radiology in particular. Imaging examinations have revolutionized the diagnosis and follow-up of cancer, facilitating its detection, staging, monitoring, and treatment^(^^1^^)^.

The ability of a radiologist to make an immediate distinction between benign and malignant bone tumors, through imaging, can guarantee an appropriate approach by qualified specialists at referral centers, ensuring excellence in the treatment of the affected patients^(^^2^^,^^3^^)^. However, inappropriate and unplanned excisions of malignant bone tumors are due to insufficient imaging or incorrect diagnostic interpretations. Therefore, in the initial evaluation of such tumors, the radiologist should communicate any suspicion of malignant potential or local aggressiveness, in order to inform the therapeutic decision-making process^(^^3^^)^.

Among the malignant bone tumors with the highest incidence in individuals ≤ 20 years of age, the most notable are osteosarcoma and Ewing’s sarcoma, the manifestations of which vary depending on the geographic origin and ethnicity of the individual in question. The initial challenge for the radiologist is to make an accurate diagnosis of such tumors, which has a direct impact on the treatment and survival of the affected patients. The first step in the imaging investigation is conventional X-ray, which can be followed by examinations of greater complexity, such as computed tomography and magnetic resonance imaging, which have the capacity to evaluate different tissues and to obtain multiplanar images^(^^4^^)^.

The development of a cognitive map for the detection of solitary bone tumors in childhood would become an essential part of clinical practice, as a complement to diagnostic imaging, allowing the correct strategic interpretation of the diagnoses made, with little error variance. It would be possible to create an application to support professionals in the area, globally, reducing iatrogenic errors and minimizing the number of inaccurate diagnoses, as well as the negative consequences thereof^(^^5^^)^.

The development of cognitive maps and the corresponding applications has been growing. That has been achieved through the use of artificial intelligence (AI), which employs computing power in the attempt to reproduce human abilities and skills in the realm of thinking and problem solving, combining scientific, mathematical, and probabilistic data.

Recent, application-mediated, digital solutions developed by AI research groups, such as Babylon Health in the United Kingdom, are widely accessible and are being used efficiently. Those systems promote quality health care by providing features such as speech recognition in the form of chat bots, aiding in the prevention and diagnosis of diseases.

Various research centers specializing in AI have developed training algorithms for specific demands adapted to the reality of each diagnostic area, as well as decision trees, such as those for skin cancer detection (mainly melanoma) and the evaluation of diabetic retinopathy-both of which have had a high level of success in comparison with that of trained physicians. In addition, the development of facial recognition software has the potential to facilitate the diagnosis of rare genetic diseases, which often go underdiagnosed due to a lack of knowledge on the part of physicians.

The diagnostic ability developed by state-of-the-art computers from a specific database, together with logical human reasoning, brings enormous efficiency to medical practice and has a major financial impact. The optimistic view of the applicability of AI in the medical field is that it will enhance, rather than replace, human performance, combining the benefit of creative innovation with the replication of an appropriate formula. In contrast, fear and insecurity about AI replacing physicians in several areas remains an unknown factor in the evaluation of this new paradigm^(^^6^^)^.

Technological advances, at the organizational or individual level, are virtually guaranteed in the near future. However, it should be borne in mind that face-to-face physician-patient interaction will continue to be essential in the diagnostic and therapeutic approach, as will the involvement of multidisciplinary teams including psychologists and physiotherapists, in order to ensure the well-being and maintain the health status of patients and their families in different areas of medicine. Therefore, it is necessary to prioritize a model of affective and emotional care based primarily on human relationships.
